# Effect of Statins on Serum level of hs-CRP and CRP in Patients with Cardiovascular Diseases: A Systematic Review and Meta-Analysis of Randomized Controlled Trials

**DOI:** 10.1155/2022/8732360

**Published:** 2022-01-28

**Authors:** Tahmineh Kandelouei, Mitra Abbasifard, Danyal Imani, Saeed Aslani, Bahman Razi, Mahdieh Fasihi, Sajad Shafiekhani, Keyhan Mohammadi, Tannaz Jamialahmadi, Željko Reiner, Amirhossein Sahebkar

**Affiliations:** ^1^Department of Oncological Sciences, Huntsman Cancer Institute, University of Utah, Salt Lake City, UT 84112, USA; ^2^Molecular Medicine Research Center, Research Institute of Basic Medical Sciences, Rafsanjan University of Medical Sciences, Rafsanjan, Iran; ^3^Department of Internal Medicine, School of Medicine, Ali Ibn Abi Talib Hospital, Rafsanjan University of Medical Sciences, Rafsanjan, Iran; ^4^Department of Immunology, School of Public Health, Tehran University of Medical Sciences, Tehran, Iran; ^5^Department of Immunology, School of Medicine, Tehran University of Medical Sciences, Tehran, Iran; ^6^Department of Hematology and Blood Transfusion, School of Medicine, Tarbiat Modares University, Tehran, Iran; ^7^Departments of Biomedical Engineering, School of Medicine, Tehran University of Medical Sciences, Iran; ^8^Research Center for Biomedical Technologies and Robotics, Tehran, Iran; ^9^Students Scientific Research Center, Tehran University of Medical Sciences, Tehran, Iran; ^10^Faculty of Pharmacy, Tehran University of Medical Sciences, Tehran, Iran; ^11^Department of Nutrition, Faculty of Medicine, Mashhad University of Medical Sciences, Mashhad, Iran; ^12^Department of Internal Medicine, University Hospital Center Zagreb, School of Medicine, University of Zagreb, Zagreb, Croatia; ^13^Applied Biomedical Research Center, Mashhad University of Medical Sciences, Mashhad, Iran; ^14^Biotechnology Research Center, Pharmaceutical Technology Institute, Mashhad University of Medical Sciences, Mashhad, Iran; ^15^Department of Medical Biotechnology, Faculty of Medicine, Mashhad University of Medical Sciences, Mashhad, Iran; ^16^Department of Biotechnology, School of Pharmacy, Mashhad University of Medical Sciences, Mashhad, Iran

## Abstract

**Background:**

Several studies have reported that statins have anti-inflammatory effects. Nevertheless, results of clinical trials concerning the effect of statins on the levels of C-reactive protein (CRP) and high-sensitivity CRP (hs-CRP) have been inconsistent. Therefore, we performed a systematic review and meta-analysis of randomized clinical trials (RCTs) evaluating the effect of statins on CRP and hs-CRP levels in patients with cardiovascular diseases (CVDs).

**Methods:**

Literature search of the major databases was performed to find eligible RCTs assessing the effect of statins on serum levels of CRP and hs-CRP from the inception until the last week of April 2021. The effect sizes were determined for weighted mean difference (WMD) and 95% confidence intervals (CI).

**Results:**

26 studies were identified (3010 patients and 2968 controls) for hs-CRP and 20 studies (3026 patients and 2968 controls) for CRP. Statins reduced the serum levels of hs-CRP (WMD = −0.97 mg/L; 95% CI: -1.26 to -0.68 mg/L; *P* < 0.001) and CRP (WMD = −3.05 mg/L; 95% CI: -4.86 to -1.25 mg/L; *P* < 0.001) in patients with CVDs. Statins decreased the serum levels of hs-CRP in patients receiving both high-intensity and moderate/low-intensity treatments with these drugs. In addition, the duration of treatment longer than 10 weeks decreased hs-CRP levels. Only high-intensity statin treatment could marginally decrease serum levels of CRP in CVDs patients.

**Conclusions:**

This meta-analysis showed the efficacy of statins to reduce the concentrations of CRP and hs-CRP in patients with different types of CVDs.

## 1. Introduction

Statins, which are 3-hydroxy-3-methylglutaryl coenzyme A (HMG-CoA) reductase inhibitors, are broadly used as lipid-lowering drugs in patients with cardiovascular diseases (CVDs) [1, 2]. Although several classes of newer agents have been introduced in recent decades, statins still remain as the cornerstone of management of dyslipidemias [3–6]. Statins interfere with cholesterol synthesis in the liver cells and, therefore, decrease the levels of total cholesterol and low-density lipoprotein cholesterol (LDL-C) in serum [7]. Statins have been associated with decreased mortality rate in patients with CVDs. On the other hand, statins have anti-inflammatory effects, and therefore, they might be beneficial in diseases related to inflammation including not only atherosclerosis [8] but also congestive heart failure (CHF) [9], nephropathy [10], central nervous system (CNS) diseases [11], autoimmune disease [12], sepsis [13], gastrointestinal diseases [14], delirium [15], COVID-19 [16], bone remodeling/osteoporosis [17], and macular degeneration [18]. However, the treatment with statins in these diseases is still not widely accepted despite a wide range of lipid-independent and pleiotropic actions [19–21].

C-reactive protein (CRP) is an acute phase protein that is part of the pentraxin protein family. It is primarily produced by liver cells and in small quantities by some other cells like vascular smooth muscle cells and macrophages. Atherosclerotic lesions also produce CRP [22]. A lot of evidence shows that inflammatory events are underlying cause in the development of cardiovascular events and atherosclerotic CVDs, such as acute coronary syndromes (ACS) [23, 24]. CRP, as a well-known marker of inflammation, has been associated with atherosclerotic plaques development as well as with destabilization of plaques and promotion of occlusive thrombi [25]. CRP has been shown to injure the glycocalyx of vascular endothelium resulting in dysfunction of endothelium which is considered to be the first step in atherogenesis [26]. CRP can induce the renin-angiotensin-aldosterone system, and it enhances the proatherogenic function of angiotensin causing functional and structural modifications of vessel walls, stiffness of vessels, vascular remodeling, and interference with the regulatory systems of blood pressure [27]. CRP also triggers the production of different matrix metalloproteinases (MMP) in macrophages and endothelial cells, and it suppresses the MMP inhibitors, causing destruction of collagen in the cap of the atherosclerotic plaque and therefore destabilization and rupture of atherosclerotic plaques [28]. Several approaches have been attempted to control inflammatory diseases by reducing the levels of serum CRP [29, 30].

Statins reduce CRP levels by different mechanisms. Several inflammatory cytokines like interleukin- (IL-) 6 are involved in the stimulation of CRP production by hepatocytes. Statins interrupt the production of IL-6 suppressing the stimulatory effect of IL-6 on the generation of CRP. It is well known that statins decrease the number of LDL particles resulting in decreased levels of highly atherogenic oxidized-LDL (ox-LDL) particles, which in turn decreases the generation of inflammatory mediators by the atherosclerotic plaques that stimulate the production of CRP. By upmodulating apoA-I, statins can suppress the expression of E-selectin, intercellular adhesion molecule-1 (ICAM-1), and vascular cell adhesion molecule-1 (VCAM-1) on the activated endothelial cells causing a decreased production of CRP-stimulating factors [31]. Moreoever, in silico findings have suggested a direct interaction between statin molecules and CRP [32].

A number of clinical trials have tried to prove the effects of statins on lowering the levels of CRP and high-sensitivity CRP (hs-CRP) in the context of preventing CVDs and cardiovascular events [33–35]. JUPITER was the first trial which prospectively assessed the effects of rosuvastatin 20 mg versus placebo on rates of cardiovascular events during a maximum follow-up of 5 years (median 1.9 years), according to on-treatment concentrations of LDL-C (≥1.8 mmol/L or <1.8 mmol/L) and hs-CRP (≥2 mg/L or<2 mg/L) showing that not only decreased LDL-C but also hs-CRP are indicators of successful treatment with a statin [36]. Rosuvastatin reduced LDL-C levels by 50% and hs-CRP levels by 37%. Additionally, the results of statin therapy (pravastatin) on 472 randomly selected participants in the Cholesterol and Recurrent Events (CARE) trial indicated that median CRP levels and the mean change in CRP decreased over time (5 years) among those allocated to pravastatin (median change, -17.4%; *P* = 0.004 and mean change, -0.07 mg/dL; *P* = 0.002). Those allocated to placebo median CRP levels and the mean change in CRP tended to increase (median change, +4.2%; *P* = 0.2 and mean change, +0.07 mg/dL; *P* = 0.04) [37]. However, the results of some studies were inconsistent and inconclusive [38]. In order to obtain a clear answer, we performed a systematic review and a meta-analysis of randomized controlled trials (RCTs) which evaluated the effects of statins in lowering CRP and hs-CRP levels in different types of CVDs, such as ACS, myocardial infarction (MI), coronary artery disease (CAD), unstable angina, heart failure, stable atherosclerotic plaque, and carotid artery stenting.

## 2. Methods

This study was performed following the Preferred Reporting Items for Systematic Reviews and Meta-Analyses (PRISMA) statement [39]. Since the study did not include any studies with human participants or animals performed by any of the authors of this article, ethical approval was not necessary. The study was designed according to the international PICOS format—Population (adults with CVDs), Intervention (statins), Comparison/Comparator (control/placebo group), Outcome (to explore whether statins change the serum level of hs-CRP and CRP), and Study design (parallel and crossover clinical trials).

### 2.1. Data Sources and Search Strategy

Online literature search was performed in MEDLINE/PubMed, Scopus, and ISI Web of Science databases, and all publications evaluating the effect of statins on hs-CRP and CRP levels in patients with CVDs were analyzed until the last week of April 2021. During systematic search, the following MeSH terms and key words were used: (statins∗ OR atorvastatin OR fluvastatin OR pravastatin OR rosuvastatin OR lovastatin OR pitavastatin OR cerivastatin OR simvastatin) AND (random∗ OR “randomized controlled trial” OR “randomized trial” OR “randomized study” OR “random number”) AND (“C-reactive protein” OR CRP OR “high-sensitivity C-reactive protein” OR “hs-CRP”) AND (“acute coronary syndromes” OR ACS OR “coronary artery disease” OR CAD OR “heart failure” OR “myocardial infarction” OR “atherosclerosis” OR “stable angina” OR “unstable angina” OR “stable atherosclerotic plaques” OR angina). In order to increase the sensitivity of the literature search, the reference lists of eligible studies were scanned manually. Furthermore, no restrictive filter was added to the search strategy.

### 2.2. Inclusion and Exclusion Criteria

Trials were included in the quantitative analysis if they met the following criteria: (1) studies with randomized controlled trial (RCT) design; (2) RCTs considering the effect of statins on hs-CRP and CRP serum level in patients with CVDs; and (3) RCTs with sufficient, calculable, or convertible data regarding the mean changes of hs-CRP and CRP, along with standard deviation (SD) for both intervention and control groups. Age, gender, dose and duration of statin therapy, and number of participants in both groups were not the cause of inclusion or exclusion of studies. The publications were excluded if they were performed on animals or *in vitro* or did not report levels of hs-CRP and CRP both at starting point and at the end of treatment. Meta-analyses, case reports, book chapters, unpublished data, and gray literature (dissertations, congress abstracts, and patents) were also excluded.

### 2.3. Study Selection and Data Extraction

Two independent authors (TK and DI) performed a systematic search and exported the results of primary search to the Endnote X9 software. Afterwards, duplicates were removed, and the other studies were assessed by title and abstract and/or full-text. Subsequently, the data of studies that fulfilled inclusion criteria were extracted separately (TK and DI) according to a predefined extraction form as follows: the first author's last name, the name of the journal, year of publication, country of origin, ethnicity, study design, mean or range of age, dosage of statin (mg/day), duration of treatment (weeks), sample size, and the mean and SD of the hs-CRP and CRP levels before and after the treatment with statins. The extracted data were compared by two authors (TK and DI), and any discrepancy was resolved by consensus, and in the case in which they did not reach a definite conclusion, the data were compared with the original article.

### 2.4. Quality Assessment

Jadad tool was used for evaluating the quality of RCTs by assessing the randomization, blinding, and dropouts (withdrawals) [40]. This scale ranges from 0 to 5, and studies with scores ≤ 2 and ≥3 were considered low- and high-quality studies, respectively.

### 2.5. Statistical Analysis

The effects of treatment with statins on hs-CRP and CRP levels were expressed by weighted mean difference (WMD), with 95% confidence interval (CI) as the continuous variable. If the outcomes were reported as the median, range, mean, standard error (SE), or CI, they were converted into the mean and standard deviation (SD). Circulating hs-CRP and CRP concentrations were expressed in mg/L. The degree of heterogeneity was measured using *I*^2^ and Cochrane's *Q* tests. Accordingly, heterogeneity was considered significant if *I*^2^ was >50% and Cochrane's *Q* was *P* < 0.10, while *I*^2^ < 50% and Cochrane's *Q* (*P* > 0.10) did not prove a heterogeneity [41, 42]. Random-effects model (REM) and fixed-effects model (FEM) were used in the presence and absence of heterogeneity, respectively. The potential publication bias was evaluated by visual inspection of asymmetry and Egger's regression test (*P* value less than 0.05 was considered significant) [43, 44]. Sensitivity analysis was used to express the impact of each publication on the pooled effect size by removing one study at a time. In order to find predefined source of heterogeneity, subgroup analyses and metaregression analyses were performed. Subgroup analyses were performed considering the dosage of statins (high intensity statin therapy and moderate/low intensity statin therapy) [45], treatment duration (>10 and ≤10 weeks), and characteristics of statins (hydrophilic and lipophilic) [46]. The nonlinear potential effects for dosage and treatment duration by statins were analyzed using fractional polynomial modeling. All statistical tests for this meta-analysis were performed with STATA statistical software (version 14.0; Stata Corporation, College Station, TX, USA) and SPSS (version 23.0; SPSS, Inc. Chicago, IL, USA).

### 2.6. Trial Sequential Analysis

In order to minimize random errors, trial sequential analysis (TSA) was performed at a level of type-I error 5%, the statistical test power (80%), relative risk reduction (RRR) (20%), and a two-sided boundary type. When the *Z*-curve crossed the trial sequential monitoring boundaries or the required information size (RIS) line, reliability of evidence was confirmed. If the *Z*-curve did not cross the trial sequential monitoring boundaries or the RIS line, more studies were implicated.

## 3. Results

### 3.1. Study Characteristics

**Figure 1:**
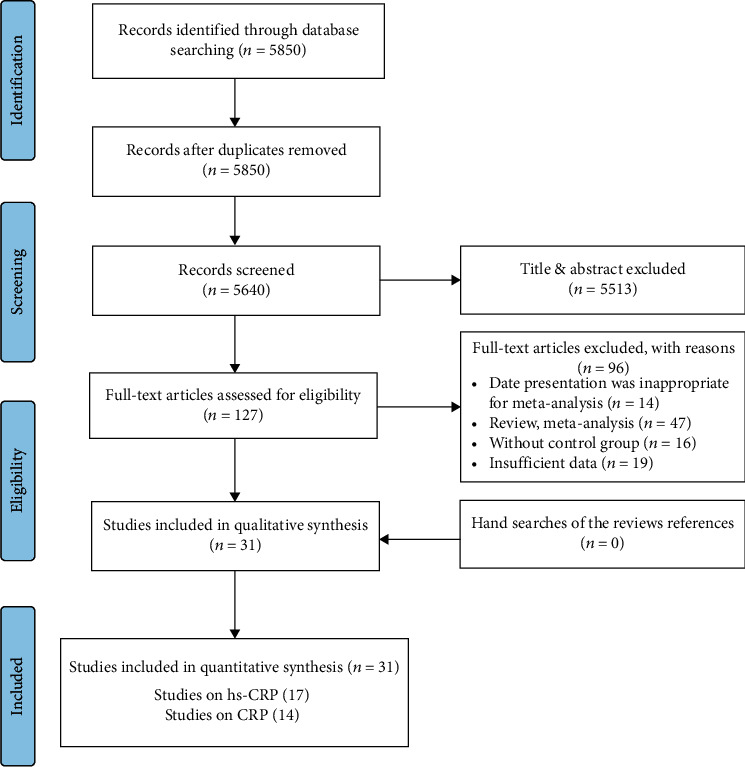
PRISMA flow diagram of the study selection process.

A total of 5850 articles were identified by the systematic literature search of databases. After excluding 210 duplicate publications and removing 5513 irrelevant publications based on titles/abstracts on 127 studies, full-text screening was performed. 96 were excluded based on the inclusion criteria because they were reviews or did not report sufficient or extractable data or did not have a control group. Finally, 31 publications were included in this meta-analysis, 17 publications (26 arms) for hs-CRP, and 14 publications (20 arms) for CRP. All included studies were performed in this century, and they were performed in different countries including China, Taiwan, Korea, Lebanon, Japan, Australia, the USA, Brazil, Argentina, the UK, Ireland, Greece, Russia, Italy, Poland, Germany, Czech Republic, and Turkey. The flow diagram of study selection is presented in Figure 1. Considering the study design, all studies were RCT. The sample size of the included studies ranged from 9 to 1216 participants, and treatment duration was between 1 to 96 weeks. Based on the Jadad scale, most of included studies received scores ≥ 3 and were considered moderate to high quality. Other characteristics of the analyzed publications are reported in Table 1.

### 3.2. Meta-Analyses of the Effect of Statins on the Serum Level of hs-CRP

**Figure 2:**
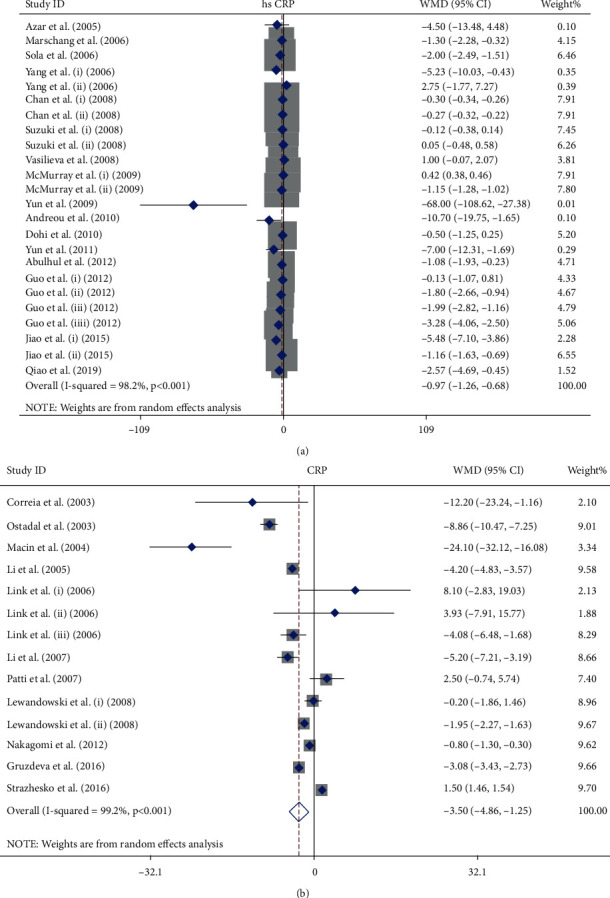
Forest plot presenting WMD and 95% CI for the effect of statin administration on hs-CRP (a) and CRP (b) levels in the overall population.

**Figure 3:**
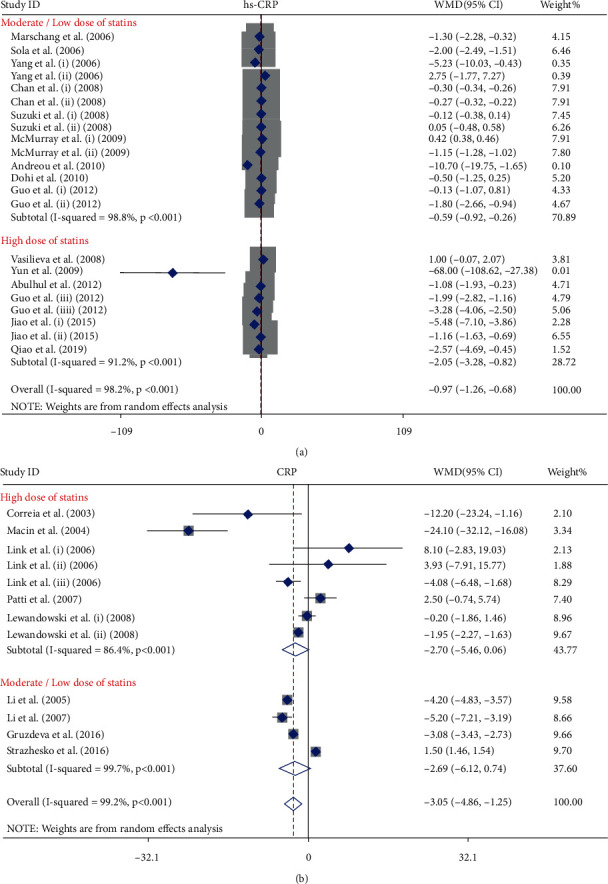
Forest plot presenting WMD and 95% CI for the effect of statin administration on hs-CRP and CRP levels in subgroup analysis by dose; (a) hs-CRP and (b) CRP.

**Figure 4:**
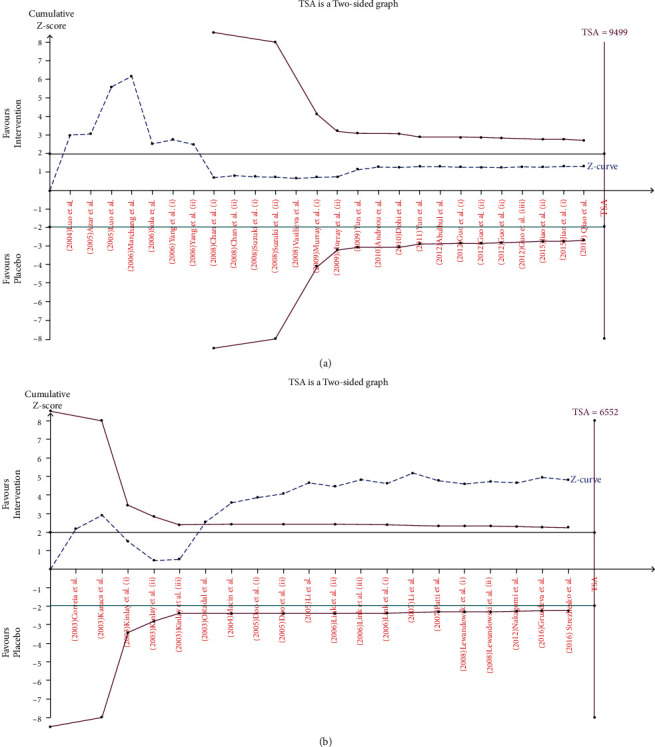
Trial sequence analysis of all the included studies for the effect of statin administration on the serum levels of hs-CRP (a) and CRP (b). Cumulative *Z*-curve (dashed blue lines), conventional boundary (green horizontal lines), trial sequential monitoring boundaries (inward sloping purple line), and required information sizes (purple vertical lines).

Seventeen publications with 26 arms including 3010 participants who received statins and 2968 participants in control groups who did not receive statins or received placebo were included in this meta-analysis. Among them, 12 publications were performed in Asian countries [47–57], 4 publications in European countries [58–61], and only one study in the USA [62]. Overall population analysis showed that statins decreased the serum level of hs-CRP (WMD = −0.97 mg/L; 95% CI: -1.26 to -0.68 mg/L; *P* < 0.001; *I*^2^: 98.2, *P*_heterogeneity_ > 0.001, Figure 2). To find more specific results, the eligible studies were stratified by dosage of statins—high-intensity statin and moderate/low-intensity statin treatment. Based on that, statins decreased the serum level of hs-CRP in both high-intensity (WMD = −2.05 mg/L; 95% CI: -3.28 to -0.82 mg/L; *P* < 0.001; *I*^2^: 91.2, *P*_heterogeneity_ > 0.001) and moderate/low-intensity groups (WMD = −0.59 mg/L; 95% CI: -0.92 to-0.26 mg/L; *P* = 0.02; *I*^2^: 98.8, *P*_heterogeneity_ > 0.001). hs-CRP level in the group that was treated with high-intensity statin therapy > 10 weeks (WMD = −2.13 mg/L; 95% CI: -3.39 to -0.86 mg/L; *P* = 0.001; *I*^2^: 86.1, *P*_heterogeneity_ > 0.001) and moderate/low-intensity statin therapy > 10 weeks (WMD = −0.57 mg/L; 95% CI: -0.90 to -0.25 mg/L; *P* = 0.001; *I*^2^: 99.1, *P*_heterogeneity_ > 0.001) (Figure 3) was also decreased when compared with the control group. These findings were not significant for treatment duration ≤ 10 weeks in both dosage groups. Unlike hydrophilic statins, lipophilic statins significantly decreased the level of hs-CRP in both high- and moderate/low-intensity statin treatment groups. The results of TSA demonstrated that the cumulative *Z*-curve crossed the conventional boundary (*P* = 0.05) which confirmed the finding of meta-analysis, but it did not cross trial sequential monitoring boundaries and did not reach the required information sizes (*n* = 9499). These findings suggested that the cumulative evidence was not enough and that more studies are required for a final conclusion (Figure 4).

### 3.3. Dose-Response Effect of Statins on hs-CRP Concentration

**Figure 5:**
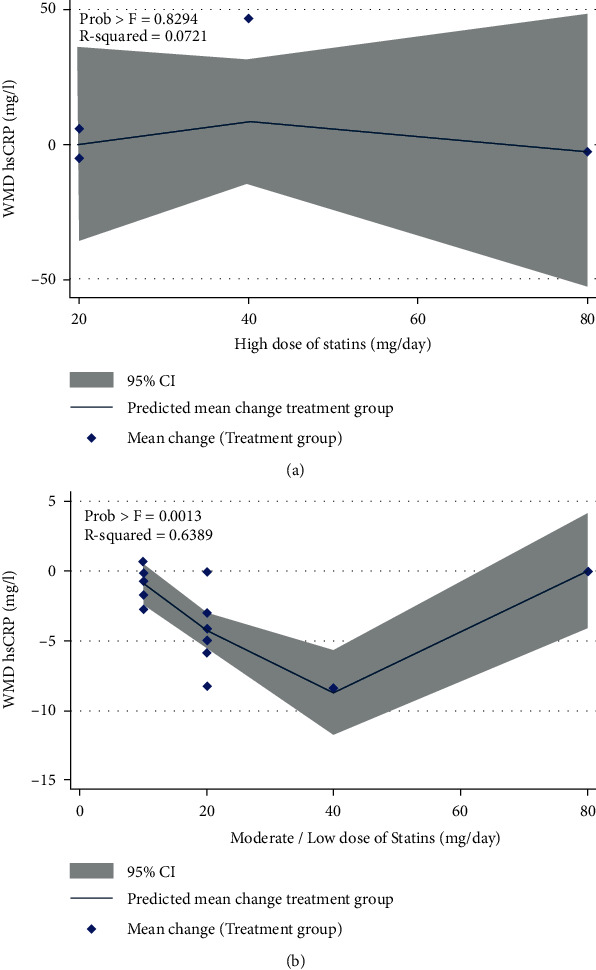
Nonlinear dose-response relations between dosage statin administration and WMD in hs-CRP: (a) high dose of statins and (b) moderate/low dose of statins.

The test for a nonlinear dose-response relationship was performed and demonstrated a nonlinear trend of moderate/low dose of statin treatment on hs-CRP concentration (Figure 5). The other dose and time response analyses could not confirm the presence of any significant trend on hs-CRP concentration (Table 2).

### 3.4. Meta-Analyses of the Effect of Statins on the Serum Level of CRP

Fourteen publications with twenty arms which included 3026 patients who were treated with statins and 2968 individuals in the control group provided data for the effect of statins on serum concentration of CRP. Among the included studies, four publications were performed on Asians [63–66], seven publications were performed on Europeans [38, 67–72], and three publications were performed on Americans [73–75]. The pooled effect indicated that treatment with statins significantly reduced CRP concentrations (WMD = −3.05 mg/L; 95% CI: -4.86 to -1.25 mg/L; *P* < 0.001; *I*^2^: 99.2, *P*_heterogeneity_ > 0.001) (Figure 2). The studies were categorized to find influence of statin dose on CRP level. High-intensity statin therapy marginally decreased serum level of CRP (WMD = −2.70 mg/L; 95% CI: -5.46 to 0.06 mg/L; *P* = 0.05; *I*^2^: 86.4, *P*_heterogeneity_ > 0.001) unlike moderate/low-intensity therapy (WMD = −2.69 mg/L; 95% CI: -6.12 to -0.74 mg/L; *P* = 0.12; *I*^2^: 99.2, *P*_heterogeneity_ > 0.001) (Figure 3). The results showed that lipophilic statins are more effective in decreasing CRP level (WMD = −3.82 mg/L; 95% CI: -7.38 to -0.26 mg/L; *P* = 0.03; *I*^2^: 90.9, *P*_heterogeneity_ > 0.001). As indicated in Figure 4, the cumulative *Z*-curve crossed the traditional boundary lines, and trial sequential monitoring boundary confirmed that the pooled result was reliable, although the cumulative sample size did not transcend the RIS.

### 3.5. Dose-Response Effect of Statins on CRP Concentration

We evaluated the dose-response relationship between the dose of statins and duration of treatment on CRP concentration. No evidence for the nonlinear relationship between CRP level and the dose of statin and duration of treatment was observed (Table 2).

### 3.6. Heterogeneity and Metaregression Analysis

**Figure 6:**
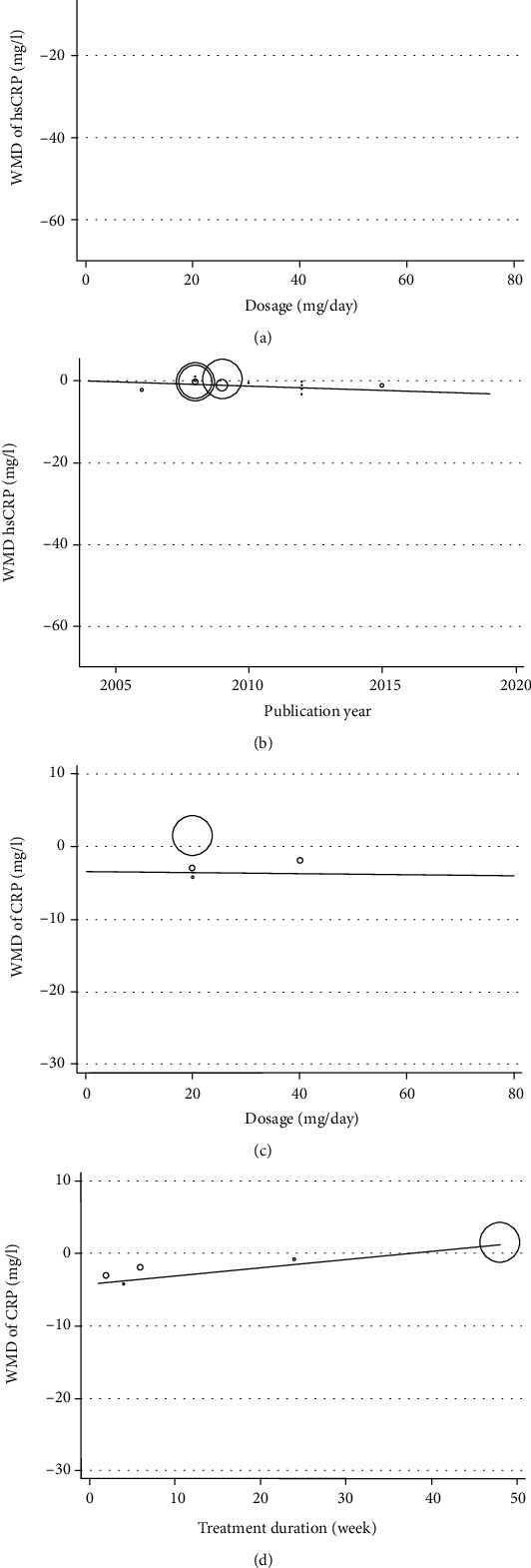
Random effects metaregression plots of the association between WMD of hs-CRP, CRP, and statin administration based on (a) hs-CRP (dosage), (b) hs-CRP (publication year), (c) CRP (dosage), and (d) CRP (treatment duration).

The degree of heterogeneity was measured by *I*^2^ and Cochrane's *Q* tests. A significant heterogeneity was observed in most of analyses. In these cases, REM was used. Metaregression analysis was used to find potential source of heterogeneity. However, it showed that the effect of statins on hs-CRP and CRP was not affected by treatment duration, dosage of statins, and publication year of the studies (Figure 6 and Table 3).

### 3.7. Publication Bias and Sensitivity Analysis

**Figure 7:**
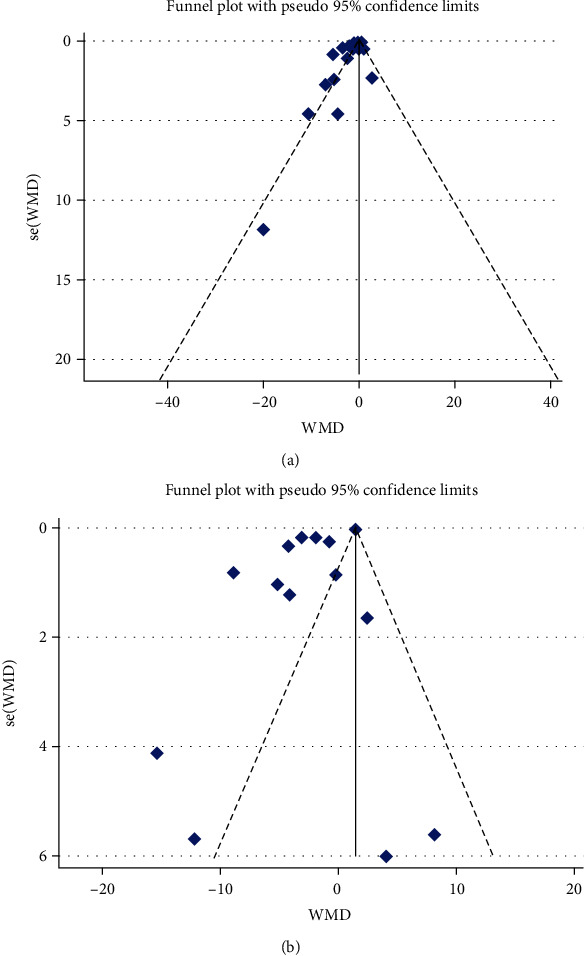
Funnel plot detailing publication biases in the studies included in the meta-analysis; (a) hs-CRP and (b) CRP.

**Figure 8:**
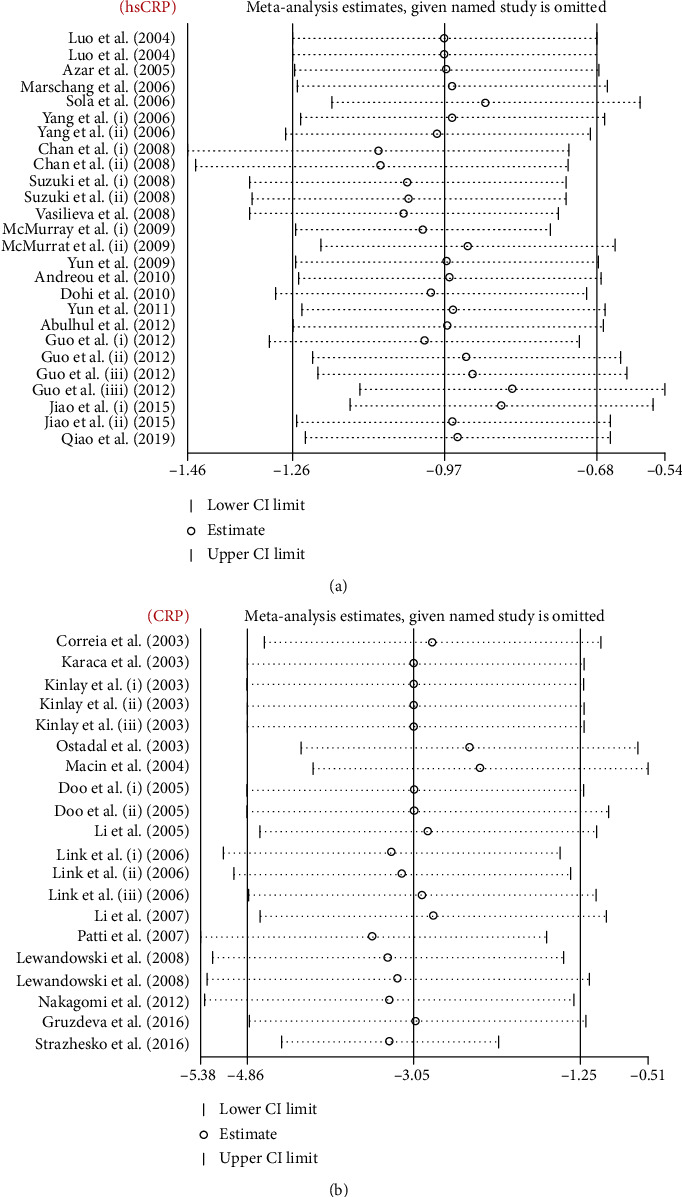
Leave-one-out sensitivity analyses of the statin therapy effect on serum hs-CRP (a) and CRP (b) level.

The result of Egger's linear regression test (hs-CRP [*z* = 1.29, *P* = 0.19] and CRP [*z* = 1.98, *P* = 0.08]) and funnel plots did not suggest any evidence of publication bias (Figure 7). In the sensitivity analysis of included studies, no individual study significantly affected the pooled effect size, proving the reliability of the results (Figure 8).

## 4. Discussion

It is well established that statins have beneficial effects on prevention of CVDs and cardiovascular events by lowering LDL-C and total cholesterol levels and having anti-inflammatory effects. Studies have proven that CRP and hs-CRP, as nonspecific markers of inflammation, are involved in the pathogenesis of CVDs. Studies have shown that reduction of LDL-C, total cholesterol levels, and markers of inflammation can reduce cardiovascular events. Elevated LDL-C is not decreased only by drugs like statins. Cicero et al. showed that red yeast rice (RYR) is among the most effective cholesterol-lowering nutraceutical which causes a reduction in LDL-C plasma levels up to 15% to 25% within 6 to 8 weeks. The decrease in LDL-C is accompanied by a proportional decrease in hs-CRP [76]. Furthermore, results of a robust meta-analysis showed that bempedoic acid is an efficient drug for lowering total cholesterol (by 15%), LDL-C (by 22.9%), apolipoprotein B (by 15.2%), and hs-CRP (by 27%) [77]. Several studies have tried to explain the role of statins in decreasing lipid levels and improving inflammation in prevention of different diseases, especially CVDs. Many of these studies had serious limitations such as little sample size, and they lacked statistical power. However, by pooling the results of different studies in a meta-analysis, it was possible to prove the effect of treatment with statins on these markers of inflammation. We performed a meta-analysis of 17 publications involving 3010 patients and 2968 controls for hs-CRP and 14 publications involving 3026 patients and 2968 controls analyzing the anti-inflammatory effects of statins by decreasing CRP and hs-CRP levels in different CVDs, including ACS, MI, CAD, unstable angina, heart failure, stable atherosclerotic plaques, and carotid artery stenting. The results indicated a significant beneficial anti-inflammatory effects of statins in patients with CVDs by decreasing the concentrations of CRP and hs-CRP in serum of these patients.

Previously, a number of meta-analyses have evaluated the effects on statins in CVDs. A most recent meta-analysis of 12 articles with 1,180 participants could not find any significant difference between patients treated with statins and those who were not treated when hs-CRP was concerned [78]. In a systematic review by Balk et al. published in 2003, statin therapy was effective in reducing levels of CRP but this effect was not dose-dependent. However, this study showed that there was no correlation between the effect of statins on CRP levels or lipids or cardiovascular outcomes [79]. Lipinski et al. in 2009 performed a meta-analysis and demonstrated that patients randomized to statins had a significant (4.2%) increase in left ventricular ejection fraction at follow-up and less hospitalizations due to deterioration of heart failure. However, treatment with statins did not affect all-cause or cardiovascular mortality in patients with heart failure [80]. A most recent study including 10,106 Finnish men without heart failure at baseline after an 8.8-year follow-up showed that several novel inflammatory biomarkers were associated with incident heart failure, suggesting early activation of respective pathways in the pathogenesis of heart failure [81]. A meta-analysis published in 2017 reported that pretreatment with statins was associated with lower levels of CRP postoperatively in patients who had coronary artery bypass graft surgery [82].

This most up-to-date meta-analysis of studies evaluating the effects of statins on CVDs indicated that statins significantly decreased levels of hs-CRP in patients with CVDs. The stratification of studies by dosage of statins suggested that statins decreased the serum levels of hs-CRP in patients who were treated with both high-intensity and moderate/low-intensity statin therapies. In addition, subgrouping based on the duration of statin treatment showed that treatment duration for more than 10 weeks decreased hs-CRP levels. Unlike hydrophilic statins, lipophilic statins significantly decreased the level of hs-CRP in both high- and moderate/low-intensity statin treatments. Regarding the influence of lipophilic statins, the findings of this meta-analysis are not in accordance with the results of Kim et al. They indicated that lipophilicity/hydrophilicity of statin did not have any effect on hs-CRP levels [83]. This study also suggests that treatment with statins significantly reduced CRP levels in CVDs patients. Considering different dosage of statins, only high-intensity statin treatment could marginally decrease the serum level of CRP in CVDs patients. Lipophilic statins were more effective in decreasing the level of CRP. The potential mechanistic role of CRP in plaque formation and subsequently an increased risk of CVDs and cardiovascular events are complex. However, theoretically, CRP could facilitate monocyte adhesion and transmigration into the vessel wall which is an early step in the atherosclerotic process. Besides, polarization of M1 macrophage by CRP is a proinflammatory trigger in plaque formation which causes macrophage infiltration in both adipose tissue and atherosclerotic lesions [84, 85].

The test for dose-response relationship between statins and levels of CRP and hs-CRP indicated a nonlinear trend of moderate/low dose of statins on hs-CRP concentration. However, no evidence for a nonlinear dose-response relationship was observed between the dose of statins and CRP level. The results of TSA for hs-CRP demonstrated that the cumulative evidence was not enough and that more studies are required for a final conclusion. Nevertheless, based on the results of TSA for CRP, trial sequential monitoring boundary indicated that the pooled result was reliable, although the cumulative sample size was lower than the expected level. Therefore, more studies are needed, at least regarding the effects of statins on hs-CRP, to prove without any doubt the beneficial effects of statins in patients with different CVDs concerning inflammatory factors.

The strength of this meta-analysis is that it included RCTs that provided the strongest level of evidence and had methodologically reliable quality. Additionally, TSA was performed for the first time in evaluating the effect of statins on CRP and hs-CRP levels and it indicated that pooled evidence concerning hs-CRP was not sufficient and that more studies are required to find a clear answer to the question about the effects of statins on hs-CRP. However, TSA results suggested that the pooled result was reliable based on the currently available data. One of the advantages of this meta-analysis was also sufficient number of studies included.

Despite our best attempt to implement a flawless systematic review and meta-analysis on the effect of statins on serum levels of hs-CRP and CRP in patients with CVDs, several limitations and caveats are still present. The first is the omission of unpublished trials with negative outcomes which is a common problem of all meta-analyses. The second problem is that heterogeneity was detected across most of our analyses. From statistical perspective, this heterogeneity describes the variability between included studies and may originate from clinical or methodological heterogeneity, from other unreported, unknown study characteristics, or may be due to chance. Therefore, to find any sources of heterogeneity and attenuate their effects, we performed a subgroup analysis and weighted meta-regression. The results of meta-regression showed that neither the treatment duration nor dosage of statins or publication year of studies was the expected source of heterogeneity. Furthermore, the other way of dealing with statistical heterogeneity, which we used in our analysis, was to incorporate a random-effects model which typically produces more conservative estimates of the significance (a wider confidence interval) since it gives proportionately higher weights to smaller studies and lower weights to larger studies than fixed effect analysis. Third, the major limitation of this study was that no data on the effects of statins on the clinical outcomes in patients with different CVDs were analyzed.

Statins have non-lipid-lowering pleiotropic effects including anti-inflammatory, reducing the levels of free fatty acids, IL-6, and PAI-1 and improving adipokine status [86–94]. Therefore, more studies exploring these effects on CVDs should be performed. The clinical outcomes of non-lipid-lowering pleiotropic effects of statins should be more thoroughly analyzed in future studies.

## 5. Conclusion

It could be concluded that this meta-analysis showed that statins can be effective in reducing CRP and hs-CRP levels in patients with CVDs, particularly those with ACS, CAD, stable atherosclerotic plaques, unstable angina, and MI. The meta-analysis showed that the current level of data for evaluating the effects of statins on CRP is almost sufficient, but further studies are required in order to clearly prove the beneficial effect of statins on hs-CRP level in patients with CVDs. Finally, although statistical analysis showed robust findings because of some limitations of the study these data should be interpreted with caution.

## Figures and Tables

**Table 1:** Demographic characteristics of the included studies.

Study author	Year	Country	Intervention/control (sample size)	Intervention (M∗/F)/control (M∗/F)	Mean age (intervention/control)	Intervention duration (week)	Target population	Dosage (mg/day)	Dosage category	Hydrophilic or lipophilic	Jadad scale	Intervention name
Hs-CRP												
Luo et al.	2004	China	11/9	M = 9, F = 2/M = 6, F = 3	67.4 ± 4.5/65.7 ± 3.6	3	AMI	20	M	Lipophile	2	Simvastatin
Luo et al.	2004	China	14/16	M = 10, F = 4/M = 13, F = 3	63.2 ± 4.2/64.6 ± 7.3	3	Unstable angina	20	M	Lipophile	2	Simvastatin
Azar et al.	2005	Lebanon	44/26	NR	63 ± 8/59 ± 1	1	CAD	NR	—	—	3	Statins
Marschang et al.	2006	Austria	47/16	M = 30, F = 17/M = 6, F = 10	61 ± 2/59 ± 2	24	CAD	NR	—	—	3	Statins
Sola et al.	2006	USA	54/54	M = 33, F = 21/M = 34, F = 20	53.8 ± 5.7/55.4 ± 6.4	96	Heart failure	20	M	Lipophile	4	Atorvastatin
Yang et al. (i)	2006	China	20/20	M = 12, F = 8/M = 13, F = 7	64.2 ± 9.7/63.5 ± 9.4	1	ACS	40	L	Lipophile	3	Fluvastatin
Yang et al. (ii)	2006	China	20/20	M = 11, F = 9/M = 13, F = 7	64.1 ± 10.9/63.5 ± 9.5	1	ACS	80	M	Lipophile	3	Fluvastatin
Chan et al. (i)	2008	Taiwan	30/30	M = 24, F = 6/M = 19, F = 11	63.77 ± 12/66.13 ± 11.50	12	CAD	10	M	Lipophile	3	Atorvastatin
Chan et al. (ii)	2008	Taiwan	30/30	M = 11, F = 9/M = 13, F = 7	63.77 ± 12/66.13 ± 11.50	24	CAD	10	M	Lipophile	3	Atorvastatin
Suzuki et al. (i)	2008	Japan	10/9	NR	71 ± 8/68 ± 7	12	CAD	10	L	Lipophile	4	Fluvastatin
Suzuki et al. (ii)	2008	Japan	10/9	NR	71 ± 8/68 ± 7	12	CAD	10	L	Hydrophile	4	Pravastatin
Vasilieva et al.	2008	Russia	19/20	NR	70.1 ± 2.4	1	ACS	40	H	Lipophile	3	Atorvastatin
McMurray et al. (i)	2009	UK	777/779	M = 578, F = 199/M = 597, F = 182	72 ± 7/73.2 ± 7.2	12	Heart failure	10	M	Hydrophile	4	Rosuvastatin hs-CRP 2.0 mg/L
McMurray et al. (ii)	2009	UK	1711/1694	M = 1319, F = 392/M = 1297, F = 397	72 ± 7/73.2 ± 7.2	12	Heart failure	10	M	Hydrophile	4	Rosuvastatin hs-CRP 2.0 mg/L
Yun et al.	2009	Korea	225/220	M = 136, F = 89/M = 137, F = 83	63 ± 11/64 ± 10	1	ACS	40	H	Hydrophile	2	Rosuvastatin
Andreou et al.	2010	Greece	21/18	M = 19, F = 2/M = 16, F = 2	66 ± 11/65 ± 11	4	Heart failure	10	M	Hydrophile	3	Rosuvastatin
Dohi et al.	2010	Japan	85/84	M = 77, F = 8/M = 69, F = 5	62.8 ± 10.4/62.3 ± 10.4	24	ACS	20	M	Lipophile	3	Atorvastatin
Yun et al.	2011	Korea	237/242	M = 130, F = 107/M = 153, F = 89	63 ± 11/63 ± 11	1	ACS	NR	—	—	3	Statins
Abulhul et al.	2012	Ireland	28/28	M = 18, F = 10/M = 20, F = 8	72 ± 9/72 ± 14	24	Heart failure	40	H	Lipophile	3	Atorvastatin
Guo et al. (i)	2012	China	47/54	M = 40, F = 7/M = 48, F = 6	62.07 ± 8.51/62.64 ± 12	24	Stable atherosclerotic plaque	10	M	Lipophile	3	Atorvastatin
Guo et al. (ii)	2012	China	45/54	M = 36, F = 9/M = 48, F = 6	62.07 ± 8.51/62.64 ± 12	24	Stable atherosclerotic plaque	20	M	Lipophile	3	Atorvastatin
Guo et al. (iii)	2012	China	43/54	M = 41, F = 2/M = 48, F = 6	62.07 ± 8.51/62.64 ± 12	24	Stable atherosclerotic plaque	40	H	Lipophile	3	Atorvastatin
Guo et al. (iiii)	2012	China	39/54	M = 34, F = 5/M = 48, F = 6	62.07 ± 8.51/62.64 ± 12	24	Stable atherosclerotic plaque	80	H	Lipophile	3	Atorvastatin
Jiao et al. (i)	2015	China	62/64	M = 40, F = 22/M = 44, F = 20	74.9 ± 8.7/75.6 ± 7.8	1	ACS	20	H	Hydrophile	2	Rosuvastatin
Jiao et al. (ii)	2015	China	62/64	M = 40, F = 22/M = 44, F = 20	74.9 ± 8.7/75.6 ± 7.8	4	ACS	20	H	Hydrophile	2	Rosuvastatin
Qiao et al.	2019	China	82/78	M = 55, F = 27/M = 50, F = 28	65.67 ± 9.14/65.08 ± 10	2	Carotid artery stenting	40	H	Lipophile	3	Atorvastatin
CRP												
Correia et al.	2003	Brazil	18/18	M = 6, F = 12/M = 9, F = 9	66 ± 9/65 ± 12	1	ACS	80	H	Lipophile	3	Atorvastatin
Karaca et al.	2003	Turkey	46/32	NR	52/52	4	CAD	20	M	Lipophile	3	Atorvastatin
Kinlay et al. (i)	2003	USA	1186/1216	M = 774, F = 408/M = 812, F = 404	64 ± 12/64 ± 11	16	ACS	80	H	Lipophile	3	Atorvastatin
Kinlay et al. (ii)	2003	USA	555/544	NR	NR/NR	16	Unstable angina	80	H	Lipophile	3	Atorvastatin
Kinlay et al.(iii)	2003	USA	631/672	NR	NR/NR	16	MI	80	H	Lipophile	3	Atorvastatin
Ostadal et al.	2003	Czech Republic	13/17	M = 11, F = 2/M = 14, F = 3	NR/NR	1	MI	0/3	—		2	Cerivastatin
Macin et al.	2004	Argentina	44/46	M = 34, F = 10/M = 33, F = 13	61.1 ± 11.5/59.3 ± 13.4	4	ACS	40	H	Lipophile	3	Atorvastatin
Doo et al. (i)	2005	China	20/30	M = 10, F = 10/M = 15, F = 15	60 ± 9/62 ± 9	1	Unstable angina	NR	—		2	Statins
Doo et al. (ii)	2005	China	20/30	M = 10, F = 10/M = 15, F = 15	60 ± 9/62 ± 9	1	Unstable angina	NR	—		2	Statins
Li et al.	2005	China	19/17	NR	NR/NR	4	Unstable angina	20	M	Lipophile	3	Atorvastatin
Link et al. (i)	2006	Germany	18/17	NR	55.5/60	1	ACS	20	H	Hydrophile	3	Rosuvastatin
Link et al. (ii)	2006	Germany	18/17	NR	55.5/60	1	ACS	20	H	Hydrophile	3	Rosuvastatin
Link et al. (iii)	2006	Germany	18/17	NR	55.5/60	6	ACS	20	H	Hydrophile	3	Rosuvastatin
Li et al.	2007	China	18/16	M = 15, F = 3/M = 13, F = 3	NR/NR	1	Unstable angina	20	M	Lipophile	3	Simvastatin
Patti et al.	2007	Italy	86/85	M = 68, F = 18/M = 67, F = 18	67 ± 10/64 ± 11	1	ACS	80	H	Lipophile	3	Atorvastatin
Lewandowski et al. (i)	2008	Poland	39/39	M = 33, F = 6/M = 33, F = 6	55 ± 9.42/54 ± 8.86	1	ACS	40	H	Lipophile	3	Atorvastatin
Lewandowski et al. (ii)	2008	Poland	39/39	M = 33, F = 6/M = 33, F = 6	55 ± 9.42/54 ± 8.86	6	ACS	40	H	Lipophile	3	Atorvastatin
Nakagomi et al.	2012	Japan	63/83	M = 44, F = 19/M = 62, F = 21	66.2 ± 11.9/64.3 ± 9.9	24	Heart failure	NR	—		2	Statins
Gruzdeva et al.	2016	Russia	60/66	M = 55, F = 5/M = 58, F = 8	56.5/60.4	2	MI	20	M	Lipophile	3	Atorvastatin
Strazhesko et al.	2016	Italy	44/38	M = 34, F = 10/M = 32, F = 6	51.4 ± 1.2/59.2 ± 1.2	48	Atherosclerosis	20	M	Lipophile	3	Atorvastatin

NR: not reported; MI: myocardial infraction; ACS: acute coronary syndrome; CAD: coronary artery disease; H: high; M: moderate; L: low; M∗: male; F: female.

**Table 2:** The effects of statin therapy on hs-CRP and CRP levels in overall population and subgroup analyses.

Parameter		Number of arms	Weighted mean difference	95% CI (*P* value)	Test of heterogeneity	
					*I* ^2^ (%)	*P*
hs-CRP						
Overall		26	-0.97	-1.26, -0.68 (<0.001)	98.2	<0.001
Dosage category						
*High intensity*		8	-2.05	-3.28, -0.82 (<0.001)	91.2	<0.001
Treatment duration (weeks)	≤10	5	-2.20	-4.59, 0.20 (0.07)	92.7	<0.001
	>10	3	-2.13	-3.39, -0.86 (0.001)	86.1	<0.001
Hydrophilicity/lipophilicity	Hydrophilic	3	-4.19	-9.10, 0.73 (0.09)	94.4	<0.001
	Lipophilic	5	-1.55	-3.02, -0.09 (0.03)	90.8	<0.001
*Moderate/low intensity*		16	-0.59	-0.92, -0.26 (0.02)	98.8	<0.001
Treatment duration (weeks)	≤10	5	-3.67	-10.94, -0.43 (0.32)	79	<0.001
	>10	11	-0.57	-0.90, -0.25 (0.001)	99.1	<0.001
Hydrophilicity/lipophilicity	Hydrophilic	4	-0.41	-1.64, 0.81 (0.50)	99.4	<0.001
	Lipophilic	11	-0.49	-0.67, -0.30 (0.001)	88.1	<0.001
CRP						
Overall		20	-3.05	-4.86, -1.25 (<0.001)	99.2	<0.001
Dosage category						
*High intensity*		11	-2.70	-5.467, 0.06 (0.05)	86.4	<0.001
Hydrophilicity/lipophilicity	Hydrophilic	3	1.19	-7.13, 9.51 (0.77)	66.8	<0.001
	Lipophilic	8	-3.82	-7.38, -0.26 (0.03)	90.9	<0.001
*Moderate intensity*		5	-2.69	-6.12, 0.74 (0.12)	99.2	<0.001

**Table 3:** Findings from metaregression analysis on the effects of statin therapy on hs-CRP and CRP levels.

Parameter	Coefficient	Standard error	95% CI	*P* value
Hs-CRP				
Dosage	-0.022	0.023	-0.071, 0.025	0.34
Treatment duration	-0.001	0.022	-0.047, 0.044	0.94
Publication year	-0.218	-1.72	-0.481, 0.045	1
CRP				
Dosage	-0.007	0.069	-0.219, 0.203	0.93
Treatment duration	0.116	0.138	-0.184, 0.417	0.84
Publication year	0.636	0.399	-0.234, 1.508	0.13
